# Cost-effectiveness of tremelimumab plus durvalumab versus sorafenib for unresectable hepatocellular carcinoma in China: an economic evaluation based on five-year overall survival data from the HIMALAYA trial

**DOI:** 10.3389/fpubh.2026.1918118

**Published:** 2026-08-27

**Authors:** Rui Feng, Ping Liang, Yajing Wang, Dong Li, Huixian Jia, Ying Zheng, Liman Huo, Qi Lv

**Affiliations:** 1 School of Disaster and Emergency Medicine, Tianjin University, Tianjin, China; 2 Department of Pharmacy, The Fourth Hospital of Hebei Medical University, Shijiazhuang, China

**Keywords:** cost-effectiveness analysis, durvalumab, health technology assessment, partitioned survival model, tremelimumab, unresectable hepatocellular carcinoma

## Abstract

**Objective:**

To assess the cost-effectiveness of tremelimumab plus durvalumab [the Single Tremelimumab Regular Interval Durvalumab (STRIDE) regimen] versus sorafenib as first-line treatment for unresectable hepatocellular carcinoma in China using 5-year follow-up data from the HIMALAYA trial.

**Methods:**

A three-state partitioned survival model comprising progression-free survival, progressed disease, and death was developed from the perspective of the Chinese healthcare system. Individual patient data were reconstructed from the published Kaplan–Meier curves from the HIMALAYA trial and used to estimate and extrapolate progression-free and overall survival. The model used 28-day cycles, a 10-year base-case time horizon, and an annual discount rate of 4.5%. Only direct medical costs were included and expressed in 2025 United States dollars. The willingness-to-pay threshold was 27,906 USD per quality-adjusted life-year (QALY). One-way sensitivity analysis, probabilistic sensitivity analysis, and scenario analyses were performed.

**Results:**

In the base-case analysis, the STRIDE regimen and sorafenib yielded 1.6937 and 1.2900 QALYs at total costs of 93,507.00 and 8,081.94 USD, respectively. The STRIDE regimen provided an additional 0.4037 QALYs at an incremental cost of 85,425.06 USD, resulting in an incremental cost-effectiveness ratio (ICER) of 211,596.11 USD/QALY. The cost of durvalumab was the principal driver of the ICER. At the prespecified willingness-to-pay threshold, the probability that STRIDE was cost-effective was 0.0%. The ICER remained above the threshold in the durvalumab patient assistance program, 15-year time-horizon, alternative overall survival extrapolation, mixture-cure, and lower progressed-disease utility scenarios. The price of durvalumab would need to decrease by 92.34% for the ICER to reach the prespecified threshold. In contrast, reducing the price of tremelimumab to zero was insufficient to lower the ICER to the threshold when the price of durvalumab remained unchanged.

**Conclusion:**

Although the STRIDE regimen improved health outcomes compared with sorafenib, it was not cost-effective at current prices in China. The findings suggest that its clinical value has not yet been matched by its economic value, primarily because of the high cumulative cost of durvalumab maintenance therapy. Future pricing, reimbursement, and patient assistance strategies should focus on reducing the cost of maintenance treatment to improve the value for money of the STRIDE regimen.

## 1 Introduction

Hepatocellular carcinoma (HCC) is a major cause of cancer morbidity and mortality worldwide and in China. According to GLOBOCAN 2022, liver cancer remains among the leading causes of cancer-related death globally (1). Recent national estimates similarly place liver cancer among the most common and fatal malignancies in China (2). Sorafenib and other molecularly targeted therapies have long been used as first-line systemic treatment for advanced or unresectable HCC, but their survival benefits are limited (3). Immune checkpoint inhibitors (ICIs), alone or in combination, have expanded the available treatment options (3). In the latest 5-year overall survival (OS) update from the HIMALAYA trial, tremelimumab plus durvalumab—the Single Tremelimumab Regular Interval Durvalumab (STRIDE) regimen—provided a durable OS benefit over sorafenib with a manageable safety profile (4). ICI-based combinations have consequently been incorporated into domestic and international treatment guidelines for HCC (3, 5).

The clinical benefits of innovative therapies are often accompanied by substantial treatment costs. In a resource-constrained healthcare system, decisions regarding access, reimbursement, and resource allocation require consideration of both incremental health benefits and incremental costs (6, 7). Previous economic evaluations of first-line immunotherapy for advanced HCC have identified drug prices, long-term survival extrapolation, and health-state utility values as important determinants of cost-effectiveness (8, 9). A localized evaluation of the STRIDE regimen using Chinese cost data and willingness-to-pay (WTP) thresholds is therefore warranted.

At the time of model development, no publicly available price for tremelimumab was available in mainland China. Ipilimumab, another cytotoxic T-lymphocyte-associated antigen 4 (CTLA-4) inhibitor with an available Chinese price, was therefore used as a proxy. This assumption was used only for model simulation and was examined in sensitivity and scenario analyses. Consistent with methodological and reporting standards for health economic modeling, uncertainty in key cost, utility, and survival-extrapolation parameters was assessed to improve the transparency and interpretability of the findings (6, 7, 10).

Using the latest 5-year OS data from the HIMALAYA trial, this study developed a partitioned survival model (PSM) to evaluate the cost-effectiveness of the STRIDE regimen versus sorafenib as first-line treatment for unresectable HCC from the perspective of the Chinese healthcare system. One-way sensitivity analysis, probabilistic sensitivity analysis, and scenario analyses were used to characterize model uncertainty. The results are intended to inform treatment selection, pricing, and reimbursement decisions for advanced HCC in China.

## 2 Materials and methods

### 2.1 Study population

The model population was based on participants in the phase III randomized HIMALAYA trial. Eligible patients were adults aged 18 years or older with unresectable HCC who had received no previous systemic anticancer therapy (11).

Patients had histologically or radiologically confirmed HCC, at least one measurable lesion according to Response Evaluation Criteria in Solid Tumors (RECIST) version 1.1, an Eastern Cooperative Oncology Group (ECOG) performance status of 0 or 1, and a Child–Pugh score of 5 or 6. These criteria defined the target population for the present first-line economic evaluation.

### 2.2 Treatment regimens

Treatment regimens were based on the phase III HIMALAYA trial and adapted to reflect Chinese clinical practice and guideline recommendations for HCC (12, 13).

First-line treatment comprised the STRIDE regimen or sorafenib. In the STRIDE group, tremelimumab was administered as a single 300-mg intravenous infusion and durvalumab as a 1,500-mg intravenous infusion every 4 weeks until disease progression, unacceptable toxicity, or withdrawal of consent. In the sorafenib group, oral sorafenib was administered at 400 mg twice daily until disease progression, unacceptable toxicity, or death.

Post-progression treatment in the HIMALAYA trial was heterogeneous and could not be fully represented by a single treatment sequence. The model therefore combined observed probabilities of subsequent treatment with standardized treatment pathways.

After progression, 40.7% of patients in the STRIDE group received subsequent systemic anticancer therapy and 59.3% received best supportive care (BSC). The corresponding proportions in the sorafenib group were 45.0 and 55.0%, respectively.

For patients receiving subsequent systemic therapy, representative regimens were selected according to Chinese clinical practice and domestic guidelines (12, 13). Lenvatinib was modeled after STRIDE and regorafenib after sorafenib. Lenvatinib was administered orally once daily at 12 mg for patients weighing at least 60 kg and 8 mg for those weighing less than 60 kg. Regorafenib was administered orally at 160 mg once daily for 21 consecutive days followed by 7 days off treatment in each 28-day cycle. Both treatments were continued until disease progression, unacceptable toxicity, or death.

Drug doses were calculated using average body-weight data from the Report on the Nutrition and Chronic Diseases Status of Chinese Residents (14) and the sex distribution reported for each HIMALAYA treatment group. The estimated weighted mean body weights were 67.82 kg for the STRIDE group and 68.18 kg for the sorafenib group.

### 2.3 Model structure

**Figure 1:**
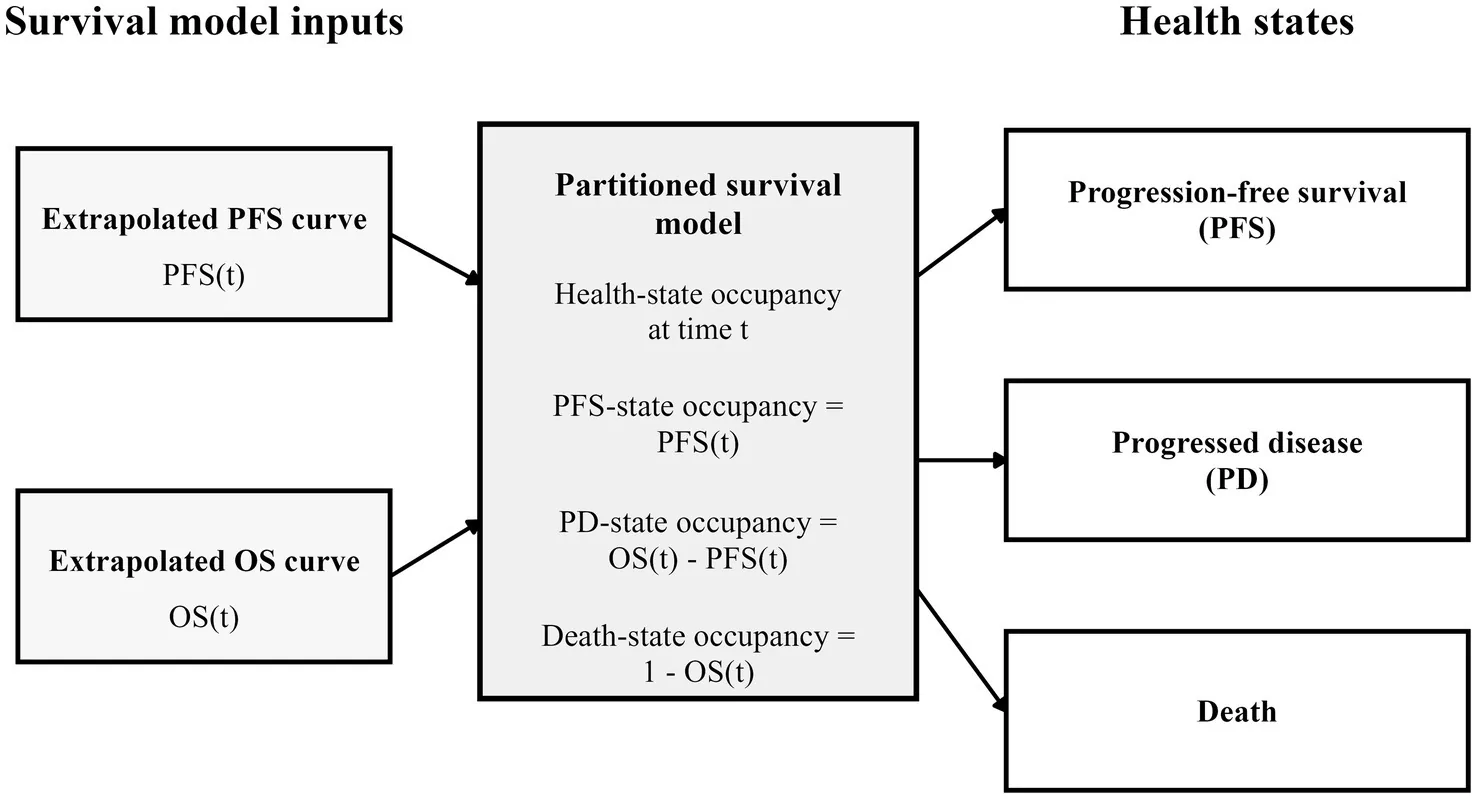
Structure of the partitioned survival model. At time *t*, the proportions of patients in the progression-free survival, progressed disease, and death states were calculated as *PFS*(*t*), *OS*(*t*) − *PFS*(*t*), and 1 − *OS*(*t*), respectively. The arrows show how health-state occupancy was derived from the extrapolated survival curves and do not represent transition probabilities.

A partitioned survival model was developed in R (version 4.4.2) with three mutually exclusive health states: progression-free survival (PFS), progressed disease (PD), and death (Figure 1).

Health-state occupancy was derived from the reconstructed OS and PFS curves from the HIMALAYA trial. The model used 28-day cycles and a 10-year base-case time horizon, which was expected to capture most clinically relevant outcomes in unresectable HCC while limiting reliance on uncertain long-term extrapolation. A 15-year scenario analysis examined the effect of a longer time horizon. Costs and health outcomes were discounted at 4.5% annually in accordance with the Guidelines for Pharmacoeconomic Evaluations in China (2025) (7).

### 2.4 Reconstruction and extrapolation of survival curves

OS and PFS data were reconstructed from the published Kaplan–Meier curves from the HIMALAYA trial (4, 11). Time and survival-probability coordinates were extracted for the STRIDE and sorafenib groups using WebPlotDigitizer. Individual patient data were reconstructed using the algorithm developed by Guyot et al. (15), together with reported sample sizes, numbers at risk, and event data. Reconstruction accuracy was evaluated by visual comparison with the published Kaplan–Meier curves and by the root mean square error (RMSE) between the extracted coordinates and reconstructed curves.

Standard parametric and flexible parametric models were fitted separately to OS and PFS data for each treatment group. Candidate models included exponential, Weibull, Gompertz, log-normal, log-logistic, gamma, and generalized gamma distributions, together with Royston–Parmar (RP) flexible parametric models using RP-hazard, RP-odds, and RP-normal specifications. Akaike information criterion, Bayesian information criterion, and RMSE values were used to identify models with acceptable within-trial fit but were not combined as the sole basis for selection. Final selection also considered visual agreement with the reconstructed Kaplan–Meier curves, consistency between OS and PFS, calibration to the observed 5-year OS rates, and the clinical plausibility of the long-term survival tails. Model-fit statistics are reported in Supplementary Table S1, and the reconstructed and extrapolated curves are shown in Supplementary Figures S1–S3.

Logical consistency between OS and PFS was required throughout the extrapolation period. PFS probabilities could not exceed OS probabilities in any cycle, thereby preventing negative occupancy of the PD state. All base-case models satisfied this constraint. In the base case, PFS was modeled using the RP-hazard model for STRIDE and the RP-normal model for sorafenib, whereas OS in both groups was modeled using the log-logistic distribution, which provided an acceptable fit and reasonably reproduced the observed 5-year OS rates.

### 2.5 Costs and utility inputs

From the perspective of the Chinese healthcare system, the model included direct medical costs only: drug acquisition, disease management, BSC, subsequent treatment, and management of adverse events (6, 7). Drug costs were based on median national tender-winning prices from January to December 2025 obtained from the Wuxu Data Platform (16).

Because no publicly available Chinese price for tremelimumab was available, the price of ipilimumab, another CTLA-4 inhibitor, was used as a proxy (17, 18, 27). This assumption approximated a potential future access price for tremelimumab in China. Although both agents block the CTLA-4 pathway, their prices are not necessarily interchangeable; uncertainty in this proxy was therefore examined in sensitivity and scenario analyses. All costs were expressed in 2025 United States dollars (USD). Costs reported in Chinese yuan were converted at the 2025 average exchange rate of 1 USD = 7.14 CNY.

Disease-management costs included laboratory tests and imaging assessments. Costs for disease management, BSC, adverse-event management, and health-state utilities were obtained from published sources. Grade 3 or higher adverse events with an incidence of at least 2% in either treatment group were included. Once an event met this criterion in one group, the observed group-specific incidence was applied to both groups, including values below 2% or zero in the other group. Adverse events were assumed to occur during the first model cycle, and incidence data were obtained from the HIMALAYA trial.

Health-state utility values were obtained from Zhao et al. (19), a China-based economic modeling study that served as the direct secondary source for the model inputs. The utility values were 0.76 for PFS and 0.68 for PD.

The WTP threshold was set at twice China’s 2025 per capita gross domestic product, equivalent to 27,906 USD/QALY, in accordance with the Guidelines for Pharmacoeconomic Evaluations in China (2025) (7). Base-case values, ranges, probability distributions, and data sources are summarized in Table 1.

**Table 1:** Input parameters used in the cost-effectiveness model.

Parameter	Description	Base value	Lower bound	Upper bound	Distribution	Source
cost_induction_exp	Tremelimumab acquisition cost per administration (STRIDE)	2,647.06	1,985.30	3,308.83	Gamma	Wuxu Data Platform
cost_maintenance_exp	Durvalumab acquisition cost per cycle (STRIDE)	7,979.65	5,984.74	9,974.56	Gamma	Wuxu Data Platform
cost_treatment_ctrl	Sorafenib acquisition cost per cycle	152.32	114.24	190.40	Gamma	Wuxu Data Platform
cost_lab	Laboratory testing cost per cycle	38.10	28.58	47.63	Gamma	(26)
cost_imaging	Imaging examination cost per cycle	38.66	29.00	48.33	Gamma	(26)
cost_PD_exp	Lenvatinib post-progression treatment cost per cycle (STRIDE)	329.28	246.96	411.60	Gamma	Wuxu Data Platform
cost_PD_ctrl	Regorafenib post-progression treatment cost per cycle (sorafenib)	106.23	79.67	132.79	Gamma	Wuxu Data Platform
cost_BSC	Best supportive care cost per cycle	357.00	267.75	446.25	Gamma	(26)
cost_EOL	End-of-life care cost per event	1,836.00	1,377.00	2,295.00	Gamma	(26)
util_PFS	Utility value for the progression-free survival state	0.76	0.61	0.91	Beta	(19)
util_PD	Utility value for the progressed disease state	0.68	0.54	0.82	Beta	(19)
discount_rate	Annual discount rate for costs and health outcomes	0.045	0	0.05	Fixed in PSA; varied in OWSA	(7)
prob_2L_exp	Probability of subsequent systemic treatment after progression (STRIDE)	0.41	0.31	0.51	Beta	(4, 11)
prob_2L_ctrl	Probability of subsequent systemic treatment after progression (sorafenib)	0.45	0.34	0.56	Beta	(4, 11)
prob_BSC_exp	Probability of best supportive care after progression (STRIDE)	0.59	0.49	0.69	Derived; not sampled	(4, 11)
prob_BSC_ctrl	Probability of best supportive care after progression (sorafenib)	0.55	0.44	0.66	Derived; not sampled	(4, 11)
cost_AE_hypertension	Management cost per event for hypertension	12.00	9.00	15.00	Gamma	(26)
cost_AE_diarrhea	Management cost per event for diarrhea	188.00	141.00	235.00	Gamma	(26)
cost_AE_PPE	Management cost per event for palmar-plantar erythrodysesthesia	15.00	11.25	18.75	Gamma	(26)
cost_AE_amylase	Management cost per event for increased amylase	44.30	33.23	55.38	Gamma	(26)
cost_AE_AST	Management cost per event for increased AST	176.55	132.41	220.69	Gamma	(26)
cost_AE_lipase	Management cost per event for increased lipase	44.30	33.23	55.38	Gamma	(26)
prob_AE_hypertension_exp	Probability of hypertension (STRIDE)	0.003	0.002	0.004	Beta	(4, 11)
prob_AE_diarrhea_exp	Probability of diarrhea (STRIDE)	0.034	0.026	0.043	Beta	(4, 11)
prob_AE_PPE_exp	Probability of palmar-plantar erythrodysesthesia (STRIDE)	0	0	0	Fixed	(4, 11)
prob_AE_amylase_exp	Probability of increased amylase (STRIDE)	0.026	0.019	0.033	Beta	(4, 11)
prob_AE_AST_exp	Probability of increased AST (STRIDE)	0.023	0.017	0.029	Beta	(4, 11)
prob_AE_lipase_exp	Probability of increased lipase (STRIDE)	0.044	0.033	0.055	Beta	(4, 11)
prob_AE_hypertension_ctrl	Probability of hypertension (sorafenib)	0.053	0.040	0.066	Beta	(4, 11)
prob_AE_diarrhea_ctrl	Probability of diarrhea (sorafenib)	0.040	0.030	0.050	Beta	(4, 11)
prob_AE_PPE_ctrl	Probability of palmar-plantar erythrodysesthesia (sorafenib)	0.088	0.066	0.110	Beta	(4, 11)
prob_AE_amylase_ctrl	Probability of increased amylase (sorafenib)	0.003	0.002	0.004	Beta	(4, 11)
prob_AE_AST_ctrl	Probability of increased AST (sorafenib)	0.016	0.012	0.020	Beta	(4, 11)
prob_AE_lipase_ctrl	Probability of increased lipase (sorafenib)	0.021	0.016	0.026	Beta	(4, 11)

AE, adverse event; AST, aspartate aminotransferase; BSC, best supportive care; PD, progressed disease; PFS, progression-free survival; PPE, palmar-plantar erythrodysesthesia; USD, United States dollar.

Within each treatment group, the probability of BSC was derived as the complement of the probability of subsequent systemic treatment: prob_BSC_exp = 1 − prob_2L_exp and prob_BSC_ctrl = 1 − prob_2L_ctrl. BSC probabilities were not varied or sampled independently. The bounds for the discount rate were used only in the one-way sensitivity analysis; the rate was fixed at 4.5% in the probabilistic sensitivity analysis.

### 2.6 Sensitivity analyses

One-way sensitivity analysis and probabilistic sensitivity analysis (PSA) were conducted to characterize parameter uncertainty. In the one-way analysis, each parameter was varied across its prespecified range while all other parameters remained fixed. Drug acquisition, disease-management, subsequent-treatment, BSC, and adverse-event management costs were varied by ±25% from their base-case values. Health-state utilities, adverse-event incidences, and subsequent-treatment probabilities were varied across their confidence intervals or plausible ranges, and the discount rate was varied from 0 to 5%. Results were presented in a tornado diagram.

PSA used 5,000 Monte Carlo simulations to evaluate joint parameter uncertainty. Gamma distributions were assigned to costs and beta distributions to utilities and probabilities. Within each treatment group, only the probability of subsequent systemic treatment was sampled; the probability of BSC was calculated as its complement so that the two probabilities summed to 1 in every simulation. To preserve clinically plausible utility ordering, any draw in which the PD utility exceeded the corresponding PFS utility was rejected and resampled. In each simulation, total costs, QALYs, and ICERs were recalculated for both treatment groups. Results were summarized using a cost-effectiveness plane and a cost-effectiveness acceptability curve (CEAC).

### 2.7 Scenario analyses

Scenario analyses assessed uncertainty related to patient assistance, drug prices, time horizons, OS extrapolation, and health-state utilities.

#### 2.7.1 Durvalumab patient assistance program scenario

The base case used the tender-winning price of durvalumab. A scenario analysis incorporated the current durvalumab patient assistance program (PAP): patients paid for the first two cycles, received two assisted cycles after approval, paid for four additional cycles, and could then receive assistance until disease progression, up to a maximum of 26 assisted cycles. This scenario estimated the effective acquisition cost under the PAP rather than the opportunity cost of donated medicines.

#### 2.7.2 Tremelimumab price variation scenario

Because tremelimumab was not marketed in China, ipilimumab was used as a price proxy. To examine uncertainty in this assumption, the tremelimumab price was varied by ±50% in both the base-case and durvalumab PAP settings, and total costs, incremental costs, incremental QALYs, and ICERs were recalculated.

#### 2.7.3 Time horizon scenario

The base-case analysis used a 10-year horizon. Because 5-year follow-up data are available and immunotherapy may confer durable survival benefits, the horizon was extended to 15 years in a scenario analysis while all other model inputs and assumptions remained unchanged.

Comparison of the 10- and 15-year results assessed the effect of truncating extrapolated long-term costs and health outcomes.

#### 2.7.4 Alternative OS extrapolation and mixture-cure scenario analyses

Structural uncertainty in long-term OS extrapolation was assessed while holding the base-case PFS models constant. The base-case log-logistic OS models were compared with log-normal, Gompertz, generalized gamma, and Royston–Parmar flexible parametric models. These alternatives had acceptable statistical fit and represented different long-term tail behaviors.

For each OS model, predicted 5-year OS was compared with the observed 5-year HIMALAYA estimates, and predicted 10-year OS was reported to characterize the extrapolated tail. Cost-effectiveness outcomes were calculated over 5-, 10-, and 15-year horizons to assess the combined effects of OS model structure and time horizon.

A mixture-cure model was also fitted separately to the OS data for each treatment group as a structural scenario. The model assumed a long-term survivor fraction and a non-cured fraction whose survival followed a parametric distribution. Costs, QALYs, incremental outcomes, and ICERs were recalculated while retaining the base-case PFS models and all other inputs.

#### 2.7.5 Lower-PD-utility scenario

Because the base-case PD utility of 0.68 may be high for progressed unresectable HCC, a scenario analysis used a value of 0.54, the lower bound reported in Zhao et al. (19). The PFS utility remained 0.76, and all other model structures and inputs were unchanged. Total and incremental QALYs and the ICER were recalculated.

#### 2.7.6 Price threshold analysis

A one-way price-threshold analysis was conducted to quantify the relationship between drug prices and the ICER and to identify the prices at which the STRIDE regimen would meet the specified WTP thresholds.

Durvalumab and tremelimumab were assessed separately with the base-case model structure unchanged. In each analysis, only the unit price of the target drug was reduced, from 0 to 100% in 1% increments; all other costs and clinical inputs remained fixed. Analyses used WTP thresholds of 27,906 and 41,859 USD/QALY, corresponding to two and three times China’s 2025 per capita GDP, respectively.

At each price level, the model was rerun and total costs, QALYs, and ICERs were recalculated. The threshold was defined as the remaining price proportion at which the ICER equaled the specified WTP threshold, with the exact crossing point estimated by linear interpolation. Reporting followed the CHEERS 2022 recommendations; the completed checklist is provided in Supplementary Table S2.

## 3 Results

### 3.1 Base-case analysis

In the base-case analysis, total costs were 93,507.00 USD for STRIDE and 8,081.94 USD for sorafenib, with corresponding outcomes of 1.6937 and 1.2900 QALYs. STRIDE provided an additional 0.4037 QALYs at an incremental cost of 85,425.06 USD, yielding an ICER of 211,596.11 USD/QALY (Table 2). Because this ICER exceeded the WTP threshold of 27,906 USD/QALY, STRIDE was not cost-effective at current prices.

**Table 2:** Base-case cost-effectiveness results.

Treatment group	Total cost (USD)	Effectiveness (QALYs)	Incremental cost (USD)	Incremental effectiveness (QALYs)	ICER (USD/QALY)
STRIDE regimen	93,507.00	1.6937	85,425.06	0.4037	211,596.11
Sorafenib	8,081.94	1.2900	—	—	—

ICER, incremental cost-effectiveness ratio; QALY, quality-adjusted life-year; STRIDE, Single Tremelimumab Regular Interval Durvalumab; USD, United States dollar. ICER was calculated using unrounded cost and QALY estimates.

### 3.2 Results of sensitivity analyses

**Figure 2:**
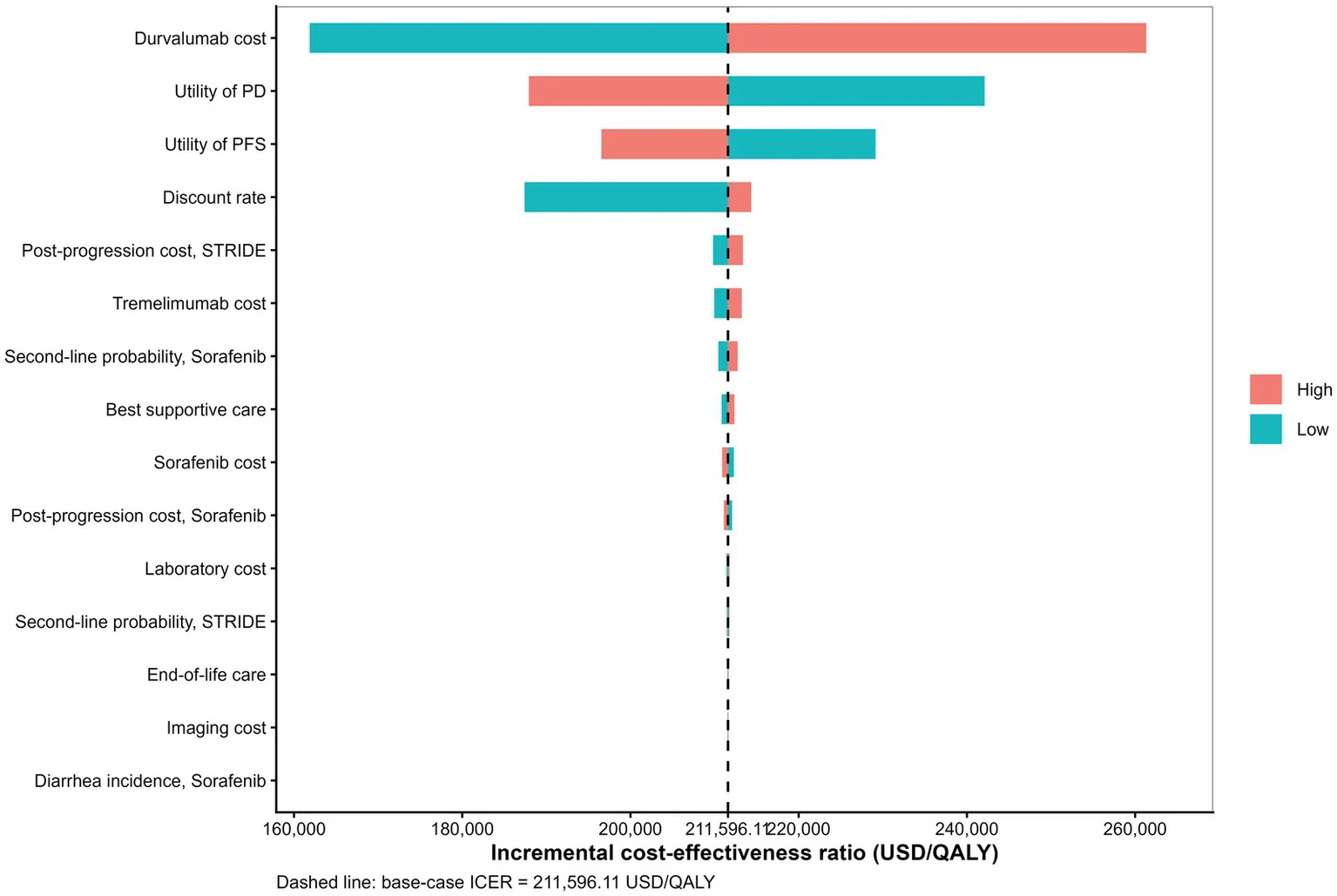
Tornado diagram for the one-way sensitivity analysis. The dashed vertical line represents the base-case ICER of 211,596.11 USD/QALY.

The one-way sensitivity analysis (Figure 2) identified the unit cost of durvalumab as the most influential parameter, followed by the utilities of the PD and PFS states and the discount rate. Laboratory testing, imaging, end-of-life care, and the incidence of diarrhea in the sorafenib group had comparatively little effect. Across the prespecified parameter ranges, the ICER remained above 27,906 USD/QALY, supporting the robustness of the base-case conclusion.

**Figure 3:**
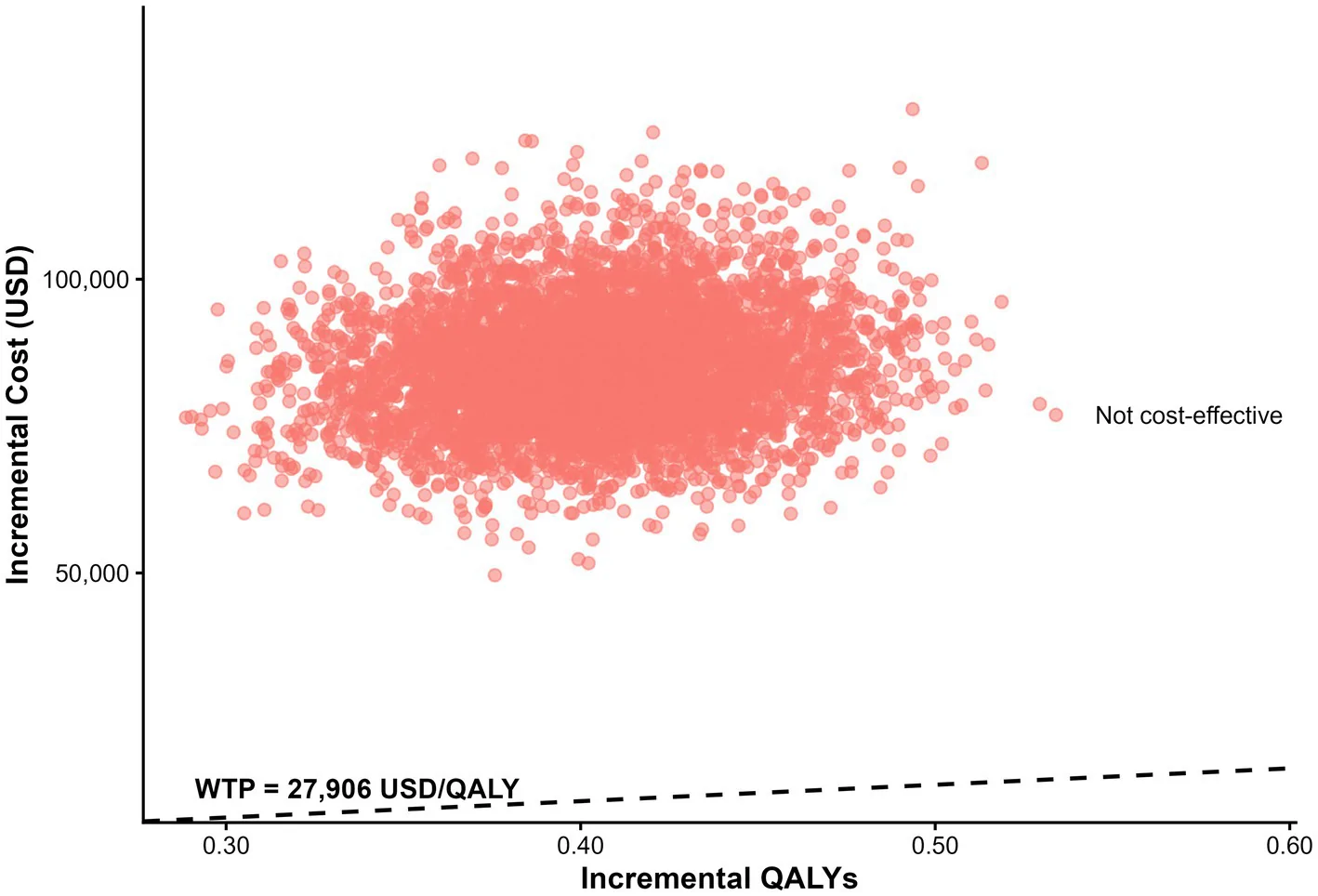
Cost-effectiveness plane from 5,000 probabilistic sensitivity analysis simulations. The dashed line represents the WTP threshold of 27,906 USD/QALY.

In the PSA with 5,000 Monte Carlo simulations, almost all incremental cost-effectiveness pairs were in the northeast quadrant of the cost-effectiveness plane (Figure 3), indicating higher costs and greater effectiveness for STRIDE than for sorafenib.

**Figure 4:**
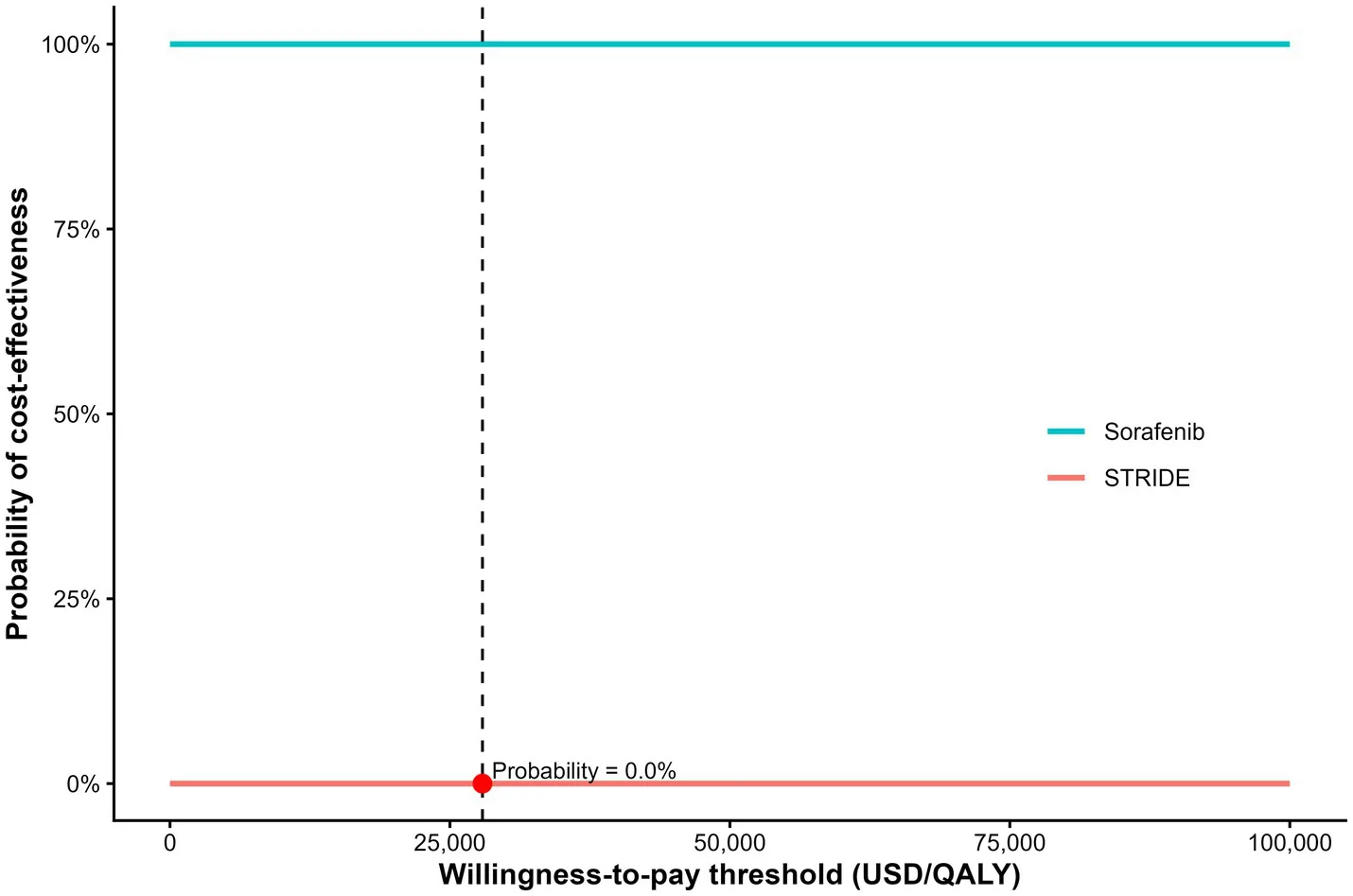
Cost-effectiveness acceptability curves for STRIDE and sorafenib. The dashed vertical line marks the WTP threshold of 27,906 USD/QALY.

The CEAC (Figure 4) showed a 0.0% probability that STRIDE was cost-effective at 27,906 USD/QALY; sorafenib was therefore the preferred strategy at this threshold. The PSA did not alter the base-case conclusion.

### 3.3 Scenario analysis results

#### 3.3.1 Durvalumab PAP scenario

Under the durvalumab PAP, total costs were 62,142.89 USD for STRIDE and 8,081.94 USD for sorafenib, with corresponding outcomes of 1.6937 and 1.2900 QALYs. STRIDE provided an additional 0.4037 QALYs at an incremental cost of 54,060.95 USD, yielding an ICER of 133,913.86 USD/QALY (Table 3). Although the PAP reduced the incremental cost and ICER relative to the base case, STRIDE remained above the WTP threshold and was not cost-effective.

**Table 3:** Cost-effectiveness results under the durvalumab patient assistance program scenario.

Treatment group	Total cost (USD)	Effectiveness (QALYs)	Incremental cost (USD)	Incremental effectiveness (QALYs)	ICER (USD/QALY)
STRIDE regimen	62,142.89	1.6937	54,060.95	0.4037	133,913.86
Sorafenib	8,081.94	1.2900	—	—	—

#### 3.3.2 Tremelimumab price variation scenario

In the base-case setting, a 50% increase in the tremelimumab price increased the STRIDE total cost to 94,830.53 USD and the incremental cost to 86,748.59 USD, yielding an ICER of 214,874.46 USD/QALY. A 50% price reduction decreased the STRIDE total cost to 92,183.47 USD and the incremental cost to 84,101.53 USD, yielding an ICER of 208,317.75 USD/QALY.

Under the durvalumab PAP, a 50% increase in the tremelimumab price produced a STRIDE total cost of 63,466.42 USD, an incremental cost of 55,384.48 USD, and an ICER of 137,186.21 USD/QALY. A 50% price reduction produced corresponding values of 60,819.36 USD, 52,737.42 USD, and 130,629.50 USD/QALY (Table 4). Total QALYs and incremental QALYs remained 1.6937 and 0.4037 because only drug price was varied. All ICERs remained above 27,906 USD/QALY.

**Table 4:** Impact of tremelimumab price variation on the cost-effectiveness of STRIDE.

Scenario	Total cost (USD)	Total QALYs	Incremental cost (USD)	Incremental QALYs	ICER (USD/QALY)
Base case (+50%)	94,830.53	1.6937	86,748.59	0.4037	214,874.46
Base case (−50%)	92,183.47	1.6937	84,101.53	0.4037	208,317.75
PAP (+50%)	63,466.42	1.6937	55,384.48	0.4037	137,186.21
PAP (−50%)	60,819.36	1.6937	52,737.42	0.4037	130,629.50

Costs and ICERs are reported to two decimal places and QALYs to four decimal places. ICERs were calculated using unrounded model outputs. ICER, incremental cost-effectiveness ratio; PAP, patient assistance program; QALY, quality-adjusted life-year; STRIDE, Single Tremelimumab Regular Interval Durvalumab. ICERs were calculated using unrounded model outputs.

#### 3.3.3 Fifteen-year scenario analysis

Over a 15-year horizon, total costs were USD 97,165.04 for STRIDE and USD 8,467.97 for sorafenib, with corresponding outcomes of 1.8422 and 1.3553 QALYs, respectively. STRIDE incurred an additional cost of USD 88,697.07 and provided an additional 0.4869 QALYs, yielding an ICER of USD 182,164.42/QALY (Table 5).

**Table 5:** Results of the 15-year scenario analysis.

Treatment group	Total cost (USD)	Effectiveness (QALYs)	Incremental cost (USD)	Incremental effectiveness (QALYs)	ICER (USD/QALY)
STRIDE regimen	97165.04	1.8422	88697.07	0.4869	182164.42
Sorafenib	8467.97	1.3553	—	—	

Costs and ICERs are reported to two decimal places and QALYs to four decimal places. ICERs were calculated using unrounded model outputs.

Extending the model time horizon reduced the ICER relative to the base-case value of USD 211,596.11/QALY; however, the ICER remained substantially above the WTP threshold of USD 27,906/QALY. Therefore, STRIDE was not cost-effective in the 15-year scenario.

#### 3.3.4 Alternative OS extrapolation and mixture-cure scenario analyses

**Figure 5:**
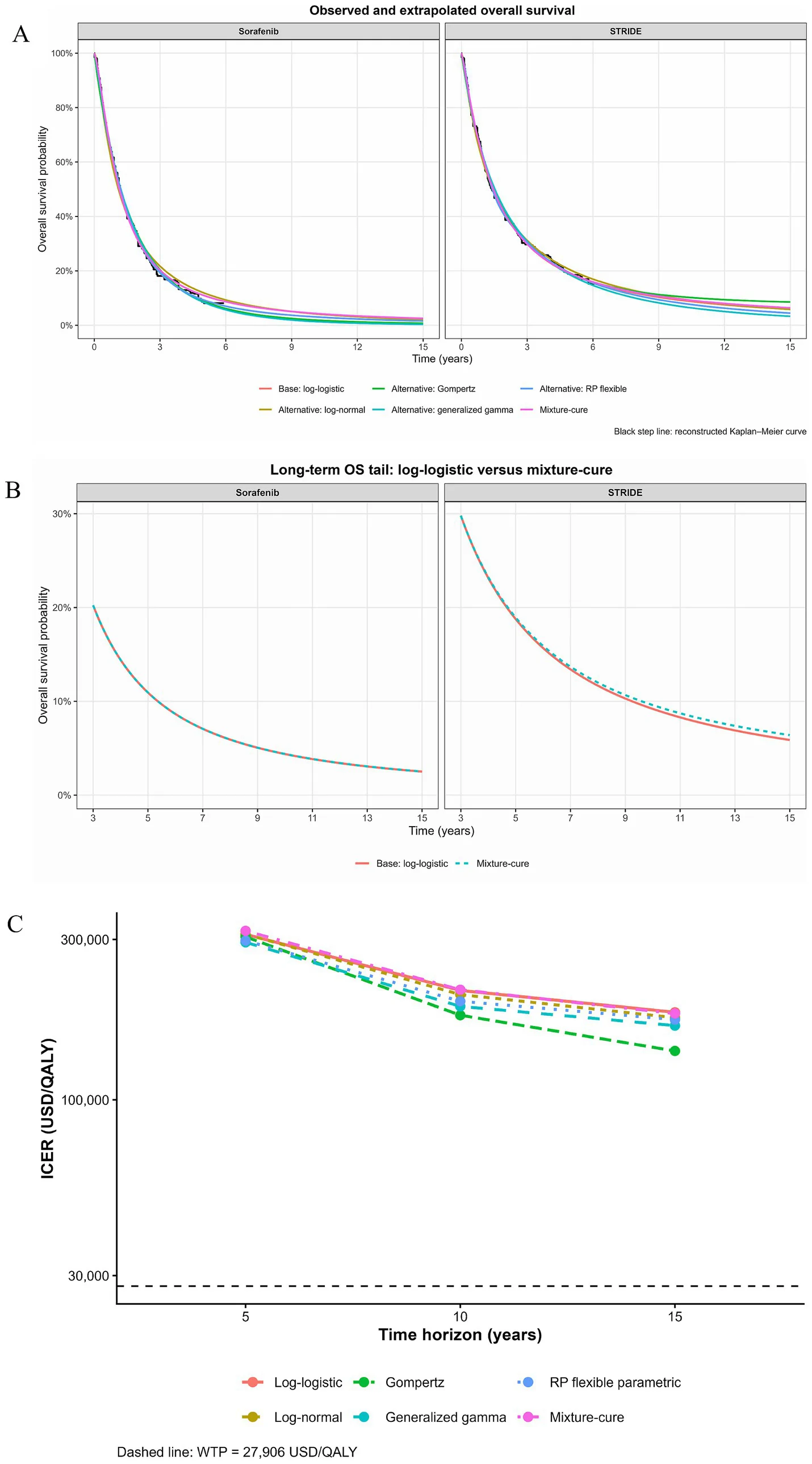
Overall survival extrapolation and cost-effectiveness across alternative model structures and time horizons. **(A)** Reconstructed Kaplan–Meier curves and fitted and extrapolated OS curves for STRIDE and sorafenib under the log-logistic, log-normal, Gompertz, generalized gamma, Royston–Parmar, and mixture-cure models over 15 years. Black step lines show the reconstructed Kaplan–Meier data. **(B)** OS tails predicted by the log-logistic and mixture-cure models from years 3 to 15. **(C)** ICERs for STRIDE versus sorafenib under each OS model over 5-, 10-, and 15-year horizons. The dashed line denotes the WTP threshold of 27,906 USD/QALY. ICER, incremental cost-effectiveness ratio; OS, overall survival; QALY, quality-adjusted life-year; STRIDE, Single Tremelimumab Regular Interval Durvalumab; WTP, willingness-to-pay.

The base-case log-logistic models predicted 5-year OS rates of 18.75% for STRIDE and 10.77% for sorafenib, compared with observed HIMALAYA rates of 19.6 and 9.4%, respectively. Across alternative models, predicted 5-year OS ranged from 18.35 to 20.22% for STRIDE and from 8.42 to 11.89% for sorafenib, indicating acceptable calibration at this landmark (Table 6, Figure 5A). Predicted 10-year OS varied from 6.93 to 10.48% for STRIDE and from 1.40 to 4.39% for sorafenib, reflecting structural uncertainty in long-term extrapolation. Within this range, the log-logistic and mixture-cure models produced similar trajectories, with modest divergence in the later STRIDE tail (Figure 5B).

**Table 6:** External validation and cost-effectiveness results under alternative OS extrapolation assumptions.

OS model scenario	STRIDE predicted 5-year OS	Sorafenib predicted 5-year OS	STRIDE predicted 10-year OS	Sorafenib predicted 10-year OS	5-year ICER	10-year ICER	15-year ICER
Log-logistic	18.75%	10.77%	9.33%	4.37%	310,784.22	211,596.11	182,164.42
Log-normal	20.22%	11.89%	9.69%	4.25%	311,187.26	205,656.36	175,515.43
Gompertz	18.83%	8.84%	10.48%	1.94%	305,539.87	178,578.82	139,782.48
Generalized gamma	18.35%	8.42%	6.93%	1.40%	294,230.43	189,703.14	166,269.36
RP flexible parametric	19.02%	9.27%	8.14%	3.11%	297,634.29	196,459.22	173,249.47
Mixture-cure	18.93%	10.94%	9.60%	4.39%	318,121.38	212,585.82	180,565.95

Observed 5-year OS rates in the updated HIMALAYA analysis were 19.6% for STRIDE and 9.4% for sorafenib. ICERs were calculated using unrounded model outputs. ICER, incremental cost-effectiveness ratio; OS, overall survival; QALY, quality-adjusted life-year; RP, Royston–Parmar; STRIDE, Single Tremelimumab Regular Interval Durvalumab.

Across the conventional and mixture-cure scenarios, ICERs ranged from 294,230.43 to 318,121.38 USD/QALY at 5 years, from 178,578.82 to 212,585.82 USD/QALY at 10 years, and from 139,782.48 to 182,164.42 USD/QALY at 15 years (Table 6, Figure 5C). The mixture-cure ICERs were 318,121.38, 212,585.82, and 180,565.95 USD/QALY at 5, 10, and 15 years, respectively. ICERs decreased as the time horizon increased but remained above 27,906 USD/QALY under every model structure.

#### 3.3.5 Lower-PD-utility scenario

In the lower-PD-utility scenario, the PD utility was reduced from 0.68 to 0.54, while the PFS utility and all other inputs remained at their base-case values. Total QALYs decreased to 1.4657 for STRIDE and 1.1129 for sorafenib, yielding an incremental gain of 0.3528 QALYs. Because costs were unchanged, the incremental cost remained 85,425.06 USD, and the ICER increased from 211,596.11 to 242,101.74 USD/QALY. The lower utility assumption did not change the cost-effectiveness conclusion (Table 7).

**Table 7:** Cost-effectiveness results under the lower-PD-utility scenario.

Scenario	PFS utility	PD utility	STRIDE total cost (USD)	STRIDE total QALYs	Sorafenib total cost (USD)	Sorafenib total QALYs	Incremental cost (USD)	Incremental QALYs	ICER (USD/QALY)
Base case	0.76	0.68	93,507.00	1.6937	8,081.94	1.2900	85,425.06	0.4037	211,596.11
Lower PD utility	0.76	0.54	93,507.00	1.4657	8,081.94	1.1129	85,425.06	0.3528	242,101.74

The PD utility was reduced from 0.68 to 0.54, the lower bound reported in Zhao et al. (19); all other inputs remained unchanged. Costs and ICERs are reported to two decimal places and QALYs to four decimal places. ICERs were calculated using unrounded model outputs.

ICER, incremental cost-effectiveness ratio; PD, progressed disease; PFS, progression-free survival; QALY, quality-adjusted life-year; STRIDE, Single Tremelimumab Regular Interval Durvalumab; USD, United States dollar.

#### 3.3.6 Price threshold analysis

**Figure 6:**
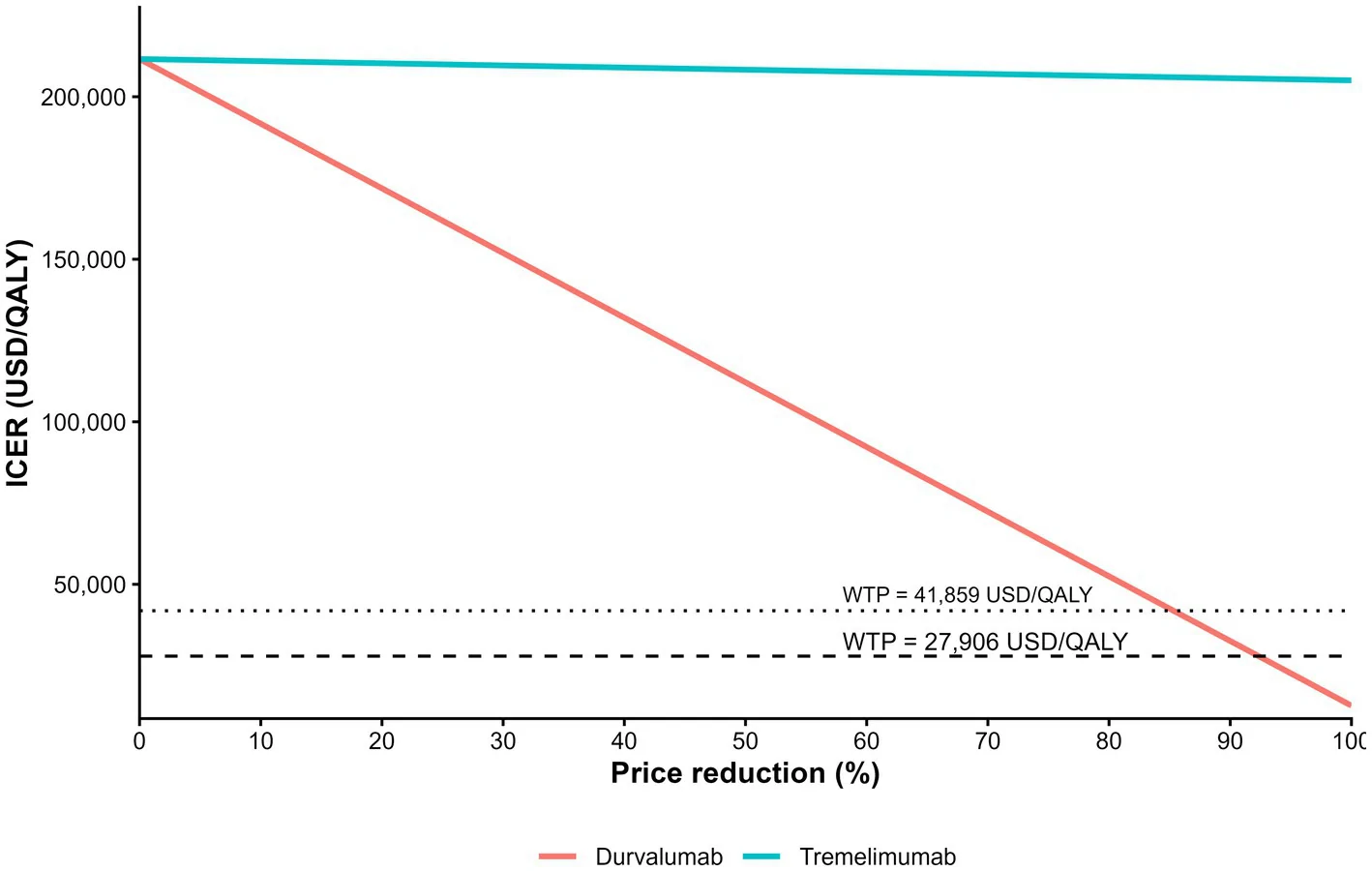
Effect of durvalumab and tremelimumab price reductions on the ICER. The horizontal axis shows the reduction from the base-case price, and the horizontal lines denote the WTP thresholds.

The price-threshold analysis showed that reductions in the prices of durvalumab and tremelimumab had markedly different effects on the ICER. To reach WTP thresholds of 27,906 and 41,859 USD/QALY, the durvalumab price would need to decrease by 92.34 and 85.32%, respectively, leaving 7.66 and 14.68% of the baseline price (Table 8, Figure 6).

**Table 8:** Price threshold results for durvalumab and tremelimumab.

Drug	WTP (USD/QALY)	Threshold price (% of baseline)	Required price reduction	Result
Durvalumab	27,906	7.66%	92.34%	Threshold reached
Durvalumab	41,859	14.68%	85.32%	Threshold reached
Tremelimumab	27,906	Not reached	Not reached	ICER remained above WTP
Tremelimumab	41,859	Not reached	Not reached	ICER remained above WTP

The threshold price is the remaining drug price expressed as a percentage of its baseline value. No threshold was identified for tremelimumab because the ICER remained above both WTP thresholds after a 100% price reduction.

ICER, incremental cost-effectiveness ratio; QALY, quality-adjusted life-year; WTP, willingness-to-pay.

No price threshold was identified for tremelimumab within the 0–100% price-reduction range. With the durvalumab price held at its base-case value, the ICER remained above both WTP thresholds even when the price of tremelimumab was reduced to zero. Reducing the price of tremelimumab alone was therefore insufficient to make STRIDE cost-effective.

## 4 Discussion

This study evaluated the cost-effectiveness of STRIDE versus sorafenib as first-line treatment for unresectable HCC using the latest long-term data from the HIMALAYA trial. Although HIMALAYA demonstrated a sustained OS benefit with STRIDE (4, 11), the base-case ICER exceeded the Chinese WTP threshold. STRIDE was therefore not cost-effective at current modeled prices. This result is consistent with previous evaluations showing that improved clinical outcomes from immunotherapy do not necessarily translate into favorable economic value when acquisition costs are high (8, 19, 20).

The results underscore the need to consider clinical benefit, affordability, and access together when assessing ICI-based regimens. Immunotherapy has expanded treatment options for unresectable HCC (21), but high acquisition costs may restrict access across payment settings. For the Chinese healthcare system, value assessment should consider incremental costs, budget sustainability, and patient financial burden in addition to survival benefits. Cost-effectiveness evidence can inform pricing, reimbursement, and resource-allocation decisions (5, 6, 20, 22).

Tremelimumab is administered once, whereas durvalumab is continued as maintenance therapy. Accordingly, the cumulative cost of durvalumab had a substantially greater effect on total costs and the ICER than the price of tremelimumab. The one-way sensitivity analysis identified the cost of durvalumab as the principal driver of the ICER, followed by the utility values for the PD and PFS states. This pattern was broadly consistent with previous China-based economic evaluations of first-line HCC treatments (23). The price-threshold analysis indicated that substantial reductions in the price of durvalumab would be required to meet the evaluated WTP thresholds. The PAP reduced the ICER but did not change the cost-effectiveness conclusion. Taken together, these findings suggest that moderate price reductions or patient-assistance arrangements alone are unlikely to make STRIDE cost-effective without a substantial reduction in the cumulative cost of maintenance therapy (19, 20, 24).

These findings may inform pricing and reimbursement decisions in China. The HIMALAYA trial demonstrated a survival benefit with STRIDE; however, its modeled economic value at current prices was limited by treatment costs. If tremelimumab enters the Chinese market, the results may inform assessments of negotiated prices, patient assistance programs, and risk-sharing arrangements. The threshold estimates should be interpreted as model-based decision points rather than predictions of future prices.

Several limitations should be considered. First, tremelimumab had no publicly available price in mainland China, and ipilimumab was therefore used as a proxy. The ipilimumab price may not represent the future market or negotiated price of tremelimumab. Although this uncertainty was explored through price-variation analyses, the scenarios could not capture all potential access arrangements, including value-based agreements or risk-sharing contracts.

Second, post-progression pathways were simplified using standardized assumptions informed by Chinese practice and guidelines. Trial-based OS and PFS implicitly reflected the heterogeneous subsequent treatments received in HIMALAYA, whereas post-progression costs were assigned using lenvatinib after STRIDE and regorafenib after sorafenib. This mismatch may have introduced uncertainty. Third, utility values were obtained from published economic studies and may not reflect health-related quality of life among Chinese patients in routine practice. Adverse-event disutilities were not included because suitable data were unavailable, which may have underestimated the effect of treatment toxicity on QALYs. Fourth, long-term survival was extrapolated beyond the observed trial period. Although multiple parametric and flexible models and a mixture-cure scenario were evaluated, structural uncertainty remained and increased in the later survival tail (25). Longer follow-up and Chinese real-world data are needed to validate the extrapolated outcomes (4, 25).

Finally, sorafenib was selected because it was the randomized comparator in HIMALAYA, allowing a direct comparison based on internally consistent and mature survival data (11). It is not, however, the only first-line option currently recommended in China; alternatives include atezolizumab plus bevacizumab, sintilimab plus a bevacizumab biosimilar, and lenvatinib (12, 13). A formal indirect comparison was not undertaken because cross-trial differences in populations, study design, follow-up, subsequent treatment, and outcome reporting could introduce substantial heterogeneity. The results should therefore be interpreted as a comparison of STRIDE with sorafenib and should not be extrapolated directly to other first-line regimens. Comparative economic evaluations based on head-to-head evidence or a robust network meta-analysis are warranted.

Overall, STRIDE’s modeled economic value in China was limited primarily by the cost of durvalumab maintenance therapy. Price negotiation, patient assistance, risk-sharing, or more precise identification of patients most likely to benefit may improve value for money.

## 5 Conclusion

At current modeled prices and the specified Chinese WTP threshold, STRIDE provided additional health benefits relative to sorafenib but was not cost-effective as first-line treatment for unresectable HCC. Its future economic value will depend largely on the cost of durvalumab maintenance therapy and the design of related payment arrangements.

## Data Availability

The reconstructed pseudo-individual patient data, model inputs, and R code used in this study are publicly available in the GitHub repository at: https://github.com/Huoliman/STRIDE-HCC-CEA. The reconstructed datasets were derived from publicly available aggregate Kaplan–Meier curves and do not contain original trial participant-level data or personal identifiers.
