# Supplementary material for: Cost-effectiveness of tremelimumab plus durvalumab versus sorafenib for unresectable hepatocellular carcinoma in China: an economic evaluation based on five-year overall survival data from the HIMALAYA trial
Source: Front Public Health. 2026 Aug 27;14:1918118. doi: 10.3389/fpubh.2026.1918118 (PMC13557980; doi:10.3389/fpubh.2026.1918118)
Supplement: Supplementary file 1 [file Data_Sheet_1.pdf]

Supplementary Materials

Supplementary Table S1. Goodness-of-fit statistics for candidate progression-free survival and overall survival models

Dataset	Model	LogL	AIC	BIC	RMSE	Base-case model
PFS–STRIDE	RP-hazard	-161.64	333.29	353.16	0.0316	Yes
PFS–STRIDE	RP-odds	-162.51	335.02	354.89	0.0279	No
PFS–STRIDE	Generalized gamma	-167.85	341.70	353.62	0.0318	No
PFS–STRIDE	RP-normal	-165.64	341.27	361.14	0.0323	No
PFS–STRIDE	Gompertz	-193.69	391.38	399.32	0.0209	No
PFS–STRIDE	Log-normal	-176.64	357.29	365.24	0.0428	No
PFS–STRIDE	Log-logistic	-178.37	360.74	368.69	0.0403	No
PFS–STRIDE	Weibull	-220.79	445.57	453.52	0.0509	No
PFS–STRIDE	Gamma	-228.45	460.90	468.85	0.0539	No
PFS–STRIDE	Exponential	-232.74	467.48	471.46	0.0564	No
PFS–sorafenib	RP-normal	-135.12	278.23	294.09	0.0185	Yes
PFS–sorafenib	RP-hazard	-142.09	292.17	308.02	0.0189	No
PFS–sorafenib	RP-odds	-139.34	286.69	302.54	0.0196	No
PFS–sorafenib	Generalized gamma	-148.60	303.20	315.09	0.0204	No
PFS–sorafenib	Gompertz	-157.47	318.94	326.86	0.0225	No
PFS–sorafenib	Exponential	-160.19	322.38	326.35	0.0233	No
PFS–sorafenib	Gamma	-158.75	321.50	329.43	0.0233	No
PFS–sorafenib	Log-normal	-162.12	328.23	336.16	0.0207	No
PFS–sorafenib	Weibull	-160.07	324.14	332.07	0.0234	No
PFS–sorafenib	Log-logistic	-141.62	287.24	295.17	0.0192	No
OS–STRIDE	Log-logistic	-582.39	1168.78	1176.73	0.0255	Yes
OS–STRIDE	RP-normal	-578.45	1170.90	1198.72	0.0263	No
OS–STRIDE	RP-odds	-579.02	1172.04	1199.86	0.0243	No
OS–STRIDE	Gompertz	-584.12	1172.23	1180.18	0.0186	No
OS–STRIDE	Generalized gamma	-584.62	1175.24	1187.16	0.0357	No
OS–STRIDE	RP-hazard	-579.79	1173.59	1201.41	0.0300	No
OS–STRIDE	Weibull	-591.46	1186.91	1194.86	0.0553	No
OS–STRIDE	Gamma	-593.64	1191.27	1199.22	0.0622	No
OS–STRIDE	Exponential	-597.23	1196.45	1200.43	0.0743	No
OS–STRIDE	Log-normal	-587.71	1179.42	1187.37	0.0236	No
OS–sorafenib	Log-logistic	-529.66	1063.32	1071.24	0.0201	Yes
OS–sorafenib	RP-normal	-524.57	1063.13	1090.88	0.0281	No
OS–sorafenib	RP-odds	-524.98	1063.95	1091.70	0.0258	No
OS–sorafenib	Gompertz	-531.25	1066.50	1074.43	0.0346	No
OS–sorafenib	RP-hazard	-524.91	1063.81	1091.56	0.0308	No
OS–sorafenib	Generalized	-529.24	1064.48	1076.37	0.0361	No

Dataset	Model	LogL	AIC	BIC	RMSE	Base-case model
	gamma					
OS–sorafenib	Exponential	-533.03	1068.05	1072.01	0.0451	No
OS–sorafenib	Gamma	-532.96	1069.93	1077.85	0.0457	No
OS–sorafenib	Weibull	-533.00	1069.99	1077.92	0.0445	No
OS–sorafenib	Log-normal	-540.20	1084.40	1092.33	0.0262	No

Model selection considered statistical fit (AIC, BIC, and RMSE), visual agreement with the reconstructed Kaplan–Meier curves, calibration to the observed 5-year OS rates from the updated HIMALAYA analysis, the clinical plausibility of the long-term survival tails, and consistency between the OS and PFS curves. The base-case models were RP-hazard for PFS in the STRIDE group, RP-normal for PFS in the sorafenib group, and log-logistic for OS in both groups. Abbreviations: AIC, Akaike information criterion; BIC, Bayesian information criterion; LogL, log-likelihood; OS, overall survival; PFS, progression-free survival; RMSE, root mean square error; RP, Royston–Parmar; STRIDE, Single Tremelimumab Regular Interval Durvalumab.

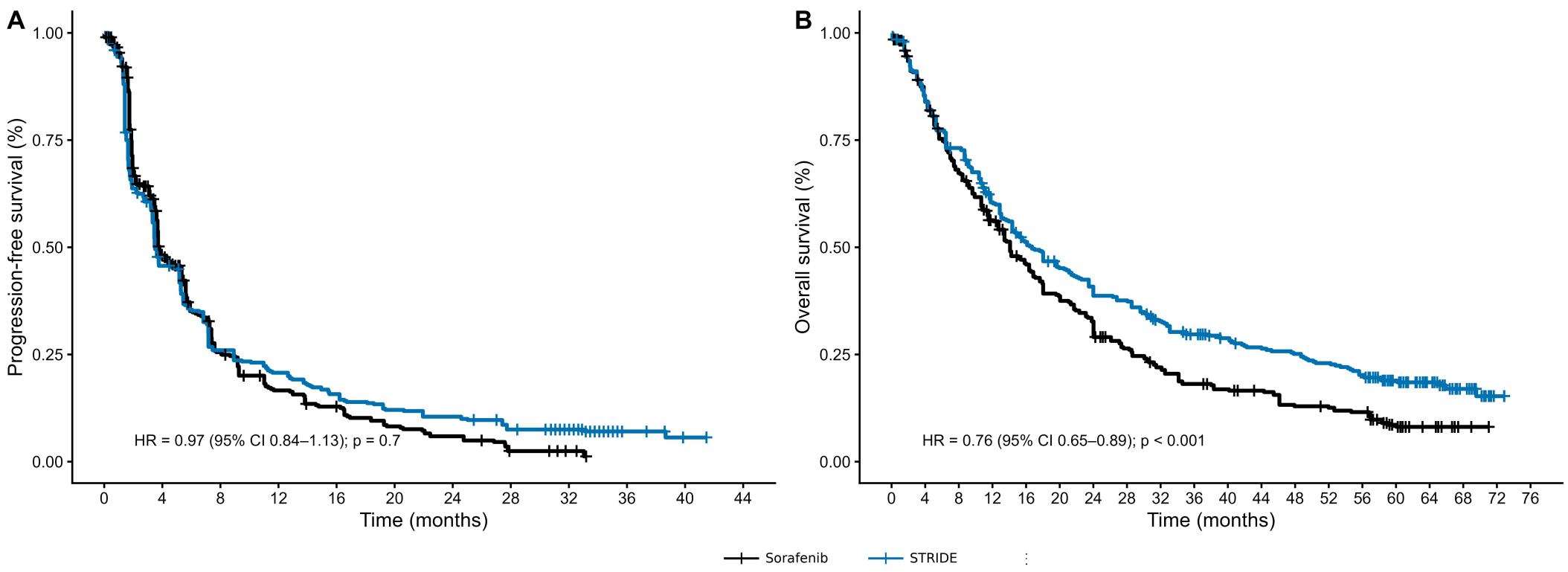

Supplementary Figure S1. Kaplan–Meier curves for progression-free survival and overall survival in the phase III HIMALAYA trial. Panel A shows progression-free survival, and Panel B shows overall survival. Black and blue curves represent sorafenib and STRIDE, respectively.

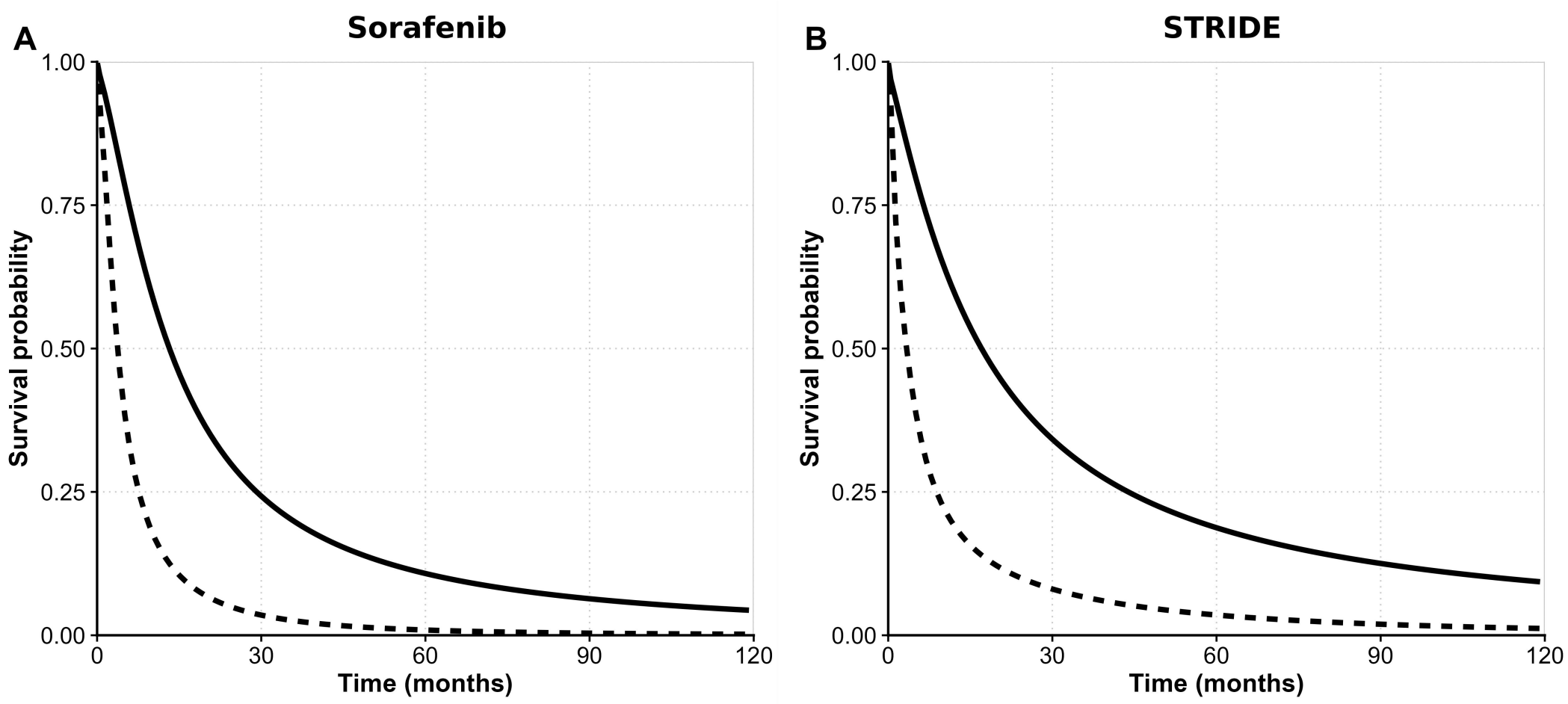

Supplementary Figure S2. Extrapolated progression-free survival and overall survival for sorafenib and STRIDE.

Panel A shows the sorafenib group, and Panel B shows the STRIDE group. Solid lines represent

overall survival, and dashed lines represent progression-free survival. The selected models were RP-normal for PFS in the sorafenib group, RP-hazard for PFS in the STRIDE group, and log-logistic for OS in both groups. For each treatment group, the OS curve remained above the PFS curve throughout the extrapolation period, with no crossing observed.

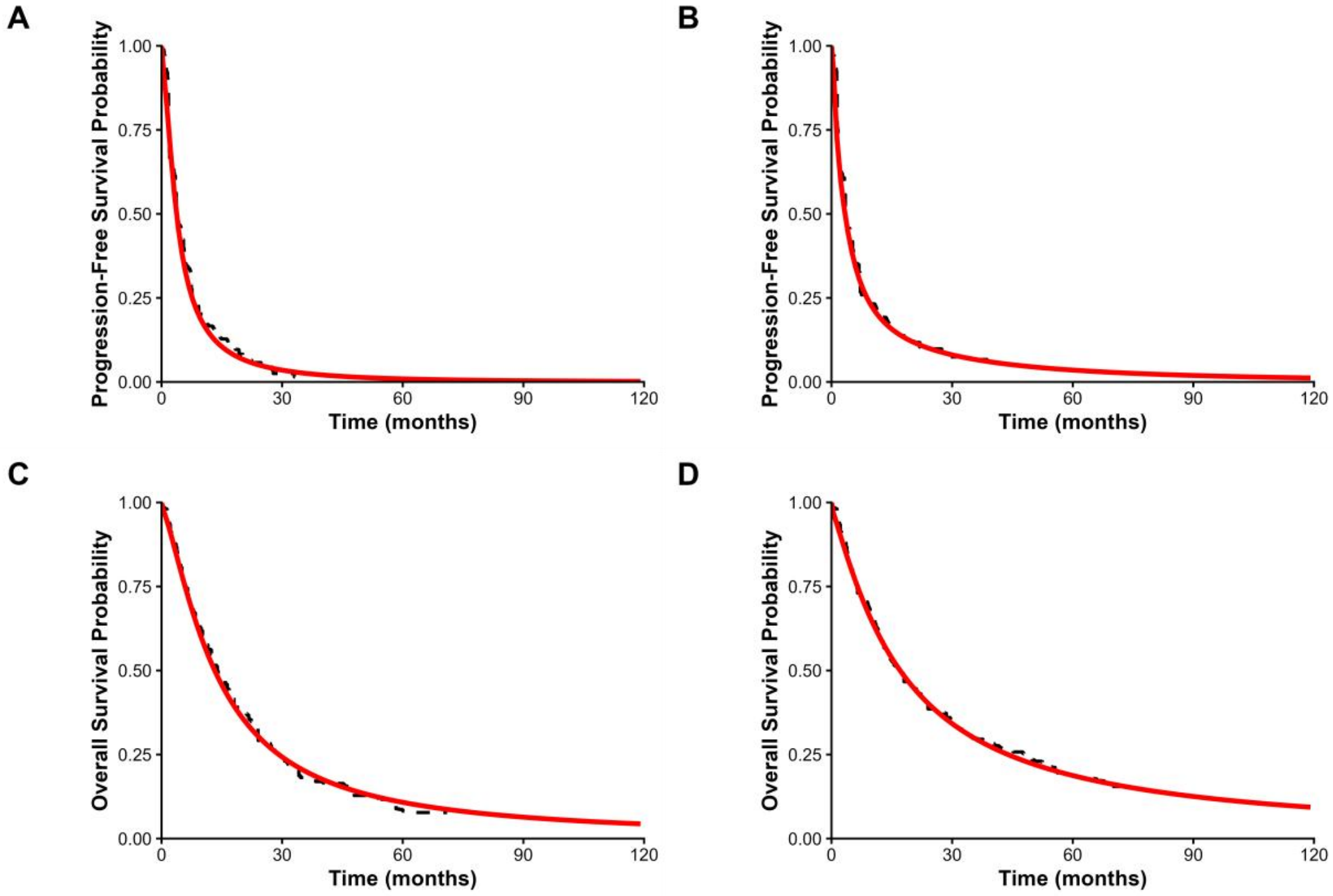

Supplementary Figure S3. Reconstructed Kaplan–Meier curves and fitted and extrapolated survival curves.

Panels A–D show the reconstructed Kaplan–Meier curves and the corresponding fitted and extrapolated survival curves. Panel A shows PFS for sorafenib, Panel B shows PFS for STRIDE, Panel C shows OS for sorafenib, and Panel D shows OS for STRIDE. Dashed black lines represent reconstructed Kaplan–Meier data, and solid red lines represent the fitted and extrapolated curves. The selected models were RP-normal for PFS in the sorafenib group, RP-hazard for PFS in the STRIDE group, and log-logistic for OS in both groups.

Supplementary Table S2. CHEERS 2022 reporting checklist

Item No.	Section	Reporting item	Location in manuscript
1	Title	Identification of the study as an economic evaluation	Title
2	Abstract	Structured summary including the population, intervention, comparator, model, costs, outcomes, ICER, uncertainty analyses, and conclusion	Abstract
3	Background and objectives	Context, rationale, and study objective	Introduction
4	Health economic analysis plan	Description of the modelling framework, data sources, and analytical approach	Methods 2.3–2.7
5	Study population	Eligibility criteria and target population	Methods 2.1
6	Setting and location	Study setting, country context, and healthcare system perspective	Introduction; Methods 2.1; Methods 2.5
7	Comparators	Description of the intervention and comparator strategies	Methods 2.2
8	Perspective	Analytic perspective used for cost estimation	Methods 2.5
9	Time horizon	Time horizon used in the base-case analysis and scenario analysis	Methods 2.3; Methods 2.7.3

Item No.	Section	Reporting item	Location in manuscript
10	Discount rate	Discount rate applied to costs and health outcomes	Methods 2.3; Methods 2.6
11	Selection of outcomes	Health outcomes used in the economic evaluation, including QALYs and ICERs	Abstract; Methods 2.5; Results 3.1
12	Measurement of effectiveness	Source and reconstruction of clinical effectiveness data	Methods 2.4
13	Measurement and valuation of preference-based outcomes	Source and values of health state utilities	Methods 2.5; Table 1
14	Measurement and valuation of resources and costs	Drug acquisition costs, disease management costs, subsequent treatment costs, BSC costs, end-of-life care costs, and adverse event management costs	Methods 2.5; Table 1
15	Currency, price date, and conversion	Currency, price year, exchange rate, and cost conversion	Methods 2.5
16	Rationale and description of model	Model type, structure, health states, cycle length, and survival-based state occupancy	Methods 2.3; Figure 1
17	Analytics and assumptions	Survival curve reconstruction, parametric and flexible survival fitting, extrapolation assumptions, and model consistency checks	Methods 2.4; Supplementary Table S1; Supplementary Figures S1–S3
18	Characterising heterogeneity	Subgroup heterogeneity was not formally assessed; scenario analyses were conducted to explore alternative assumptions	Methods 2.7; Results 3.3
19	Characterising distributional effects	Distributional effects across population subgroups were not assessed	Not applicable
20	Characterising uncertainty	One-way sensitivity analysis, probabilistic sensitivity analysis, and scenario analyses	Methods 2.6–2.7; Results 3.2–3.3
21	Approach to engagement with patients and others affected by the study	Patient or public engagement was not conducted because this was a model-based economic evaluation using published trial and cost data	Not applicable
22	Study parameters	Base-case values, ranges, probability distributions, and data sources for model parameters	Methods 2.5; Table 1; Supplementary Table S1
23	Summary of main results	Base-case total costs, QALYs, incremental costs, incremental QALYs, and ICER	Results 3.1; Table 2
24	Effect of uncertainty	Results of one-way sensitivity analysis, probabilistic sensitivity analysis, and scenario analyses	Results 3.2–3.3; Figures 2–6; Tables 3–8
25	Effect of engagement with patients and others affected by the study	Patient or public engagement was not conducted; therefore, no engagement-related results were reported	Not applicable
26	Study findings, limitations, generalisability, and current knowledge	Interpretation of findings, comparison with previous literature, policy implications, limitations, and generalisability	Discussion
27	Source of funding	Funding sources supporting the study	Funding
28	Conflict of interest	Author conflict of interest disclosures	Declarations: Conflicts of interest
